# Spider Neurotoxins as Modulators of NMDA Receptor Signaling

**DOI:** 10.1007/s12017-021-08692-w

**Published:** 2021-09-25

**Authors:** Artur Pałasz, Marek Krzystanek

**Affiliations:** 1 Department of Histology, Faculty of Medical Sciences, Medical University of Silesia, ul. Medyków 18, 40-752, Katowice, Poland; 2 Department of Psychiatry and Psychotherapy, Clinic of Psychiatric Rehabilitation, Faculty of Medical Sciences in Katowice, Medical University of Silesia, ul. Ziolowa 45/47, 40-635 Katowice, Poland

**Keywords:** NMDA, Neurotoxins, Ctenitoxin, Argiotoxin, Phoneutria

## Abstract

Molecules that selectively act on N-methyl-D-aspartate (NMDA) receptors may have a multidirectional effect by modulating the activity of NMDARs, affecting their active sites as well as by changing the composition of their subunits. The results of the clinical trials conducted so far in mood disorders and schizophrenia indicate that such agents may become new effective drugs for the treatment of these diseases. Number of spider neurotoxins e.g. ctenitoxins extracted from *Phoneutria sp*. venom act as potent and selective NMDAR blockers that do not disturb cortical and hippocampal glutamate signaling, LTP generation and synaptic neurochemistry. Possibly this intriguing kind of promising neuroregulatory peptides and polyamines can be clinically applicable in a wide spectrum of neuropsychiatric disorders, including epilepsy, neurotrauma and ischemic injuries. These novel medications can potentially be helpful in the future treatment of stroke and several neurodegenerative diseases.

## Introduction

Glutamate neurotransmission plays pivotal role in the integration and execution of higher mental functions of the brain being critically involved in the molecular mechanisms of memory, learning, consciousness and emotions (Dubois & Liu, 2021; Krzystanek & Pałasz, 2019). Among several known glutamate receptors, the ionotropic N-methyl-D-aspartate receptor (NMDAR) is considered to hold a dominant position in the generation of synaptic plasticity and hippocampal long-term potentiation (LTP). Unlike metabotropic G-coupled glutamate receptors transmembrane NMDAR molecule is a heterotetramer that consists of two obligatory GluN1 subunits and two GluN2 subunits of the same of different subtypes (GluN2A and GluN2B). GluN2 subunit may be also replaced by NR3. Glycine and glutamate binding sites are located in the homologous domains of GluN1 and GluN2A/B subunits, respectively. Several isoforms of GluN1 and at least four classes of GluN2 subunit (A-D) are currently known. The GluN1 subunit does exhibit distinct expression in majority of brain structures Psychopharmacomodulation of NMDARs is determined mainly by GluN2 subunits and their diverse isoforms may differentially affect receptor action. NMDAR activation requires uniquely simultaneous glutamate binding to GluN2 subunit, postsynaptic membrane depolarization to remove magnesium ions from the channel pore and glycine or D-serine binding to GluN1. The opening of nonselective cation channel enables sodium and calcium ions influx to neuroplasm that triggers activation of adenylate cyclase, Ca^2+^ and calmodulin-dependent protein kinase II (CaMKII). Several regulatory domains of the GluN2 subunit can bind a lot of diverse endo- and exogenous factors, including drugs and toxins e.g., polyamines, protons, zinc ions, glutathione, neurosteroids, ifenprodil, eliprodil, or even haloperidol. A common property of all NMDARs is binding several, psychomimetic open channel blockers (OCB)—ketamine, esketamine, phencyclidine (PCP), and dizocilpine (Regan et al., 2015).

Experimental and clinical studies indicate that substances that modulate NMDA receptor activity may be effective in the treatment of psychiatric diseases such as recurrent depression, bipolar depression and schizophrenic disorders. For this reason, it was decided to describe spider venom-derived NMDA receptor modulators as novel, intriguing and so far understudied agents with a putative pharmacological potential.

## Pharmacomodulation of NMDARs Function in Neuropsychiatry

Glutamate-mediated neuroplasticity plays an important role in the pathophysiology of mental disorders. NMDARs have been shown to be targets in the treatment of depressive, bipolar and schizophrenic disorders. It has been known for many years that ketamine, by blocking at the phencyclidine site within the NMDAR ion channel, rapidly improves depressive symptoms in bipolar depression (Zarate et al., 2012) as well as in the treatment of resistant bipolar depression (Diazgranados et al., 2010). Importantly, NMDAR antagonists may also reduce the number and severity of suicidal ideation in depression (Price et al., 2014). For this reason, new non-ketamine NMDAR antagonists are being searched and tested, which may become new drugs for the treatment of depressive disorders in the future (Kishimoto et al., 2016).

Contrary to the beneficial effects of NMDAR antagonists in depressive syndrome, they exert a psychodysleptic effect in schizophrenia. Therefore, in the treatment of schizophrenic disorders, substances are sought that modulate the activity of NMDARs by increasing its activity. Such NMDA receptor-enhancing agents in schizophrenia may be effective in improving common symptoms of disease, including cognitive impairment (Chang et al., 2019). Examples of such agents are N-methyl-glycine (sarcosine) and memantine—both substances by modulating NMDA receptor activity can improve the activity of NMDARs in schizophrenia and reduce schizophrenic symptoms (Andrade 2017, Chang et al., 2019, Marchi et al., 2021). Although the previous studies and meta-analyzes do not confirm the significant effectiveness of substances increasing the activity of NMDARs on cognitive disorders in schizophrenia (apart from N-acetyl cysteine), they indicate the direction of searching for new drugs to improve the effectiveness of schizophrenia pharmacotherapy (Marchi et al., 2021, Andrad, 2017, Chang et al., 2019).

Novel drugs for the treatment of schizophrenia symptoms and mood disorders may act not only on the ligand binding sites on the NMDAR, but may also alter the composition of the NMDAR subunits and thus modulate its activity. It was recently proven that antipsychotic drugs decrease the activity of NMDARs by reducing the number of GluN2B subunits in the receptor molecule (Krzystanek & Pałasz, 2019). This indicates the possibility of a multidirectional pharmacological effect on the activity and composition of NMDAR, which may be used in the development and research of new drugs selectively acting on NMDARs for the pharmacotherapy of mental disorders.

## Translational Models of Neurotoxins Applicability

Despite their high molecular specificity majority of neurotoxins can not cross blood–brain barrier that limits their potential clinical applicability in the amelioration of psychiatric diseases. Indeed, none of the described neurotoxins have been clinically tested so far, however, there are some observations that may indicate certain possibilities of their clinical application. Acyl polyamine toxin JSTX-3 or its derivatives may become new antiepileptic drugs in the future. This toxin was tested on human hippocampal slices taken surgically from patients with refractory medial temporal lobe epilepsy. The epileptiform activity induced by Mg^2+−^free artificial cerebrospinal fluid and N-methyl-D-aspartate were blocked by incubation with JSTX-3 indicating its antiepileptic effect (Salamoni et al., 2005). Also, a-Agatoxin-489 and its derivatives may be related to epilepsy treatment and may be candidates for new antiepileptic drugs with selective effect on excitatory synaptic transmission related to NMDA receptor-mediated excitatory postsynaptic currents. The action of agatoxin has been shown to be related to the antiepileptic mechanism of levetiracetam in granule cells in dentate gyrus in brain slice preparations from Wistar rats (Lee et al., 2009). As mentioned earlier, argiotoxins, agatoxins and neurotoxin JSTX-3 might become new non-ketamine NMDAR antagonists to tested in the treatment of depression as new antidepressants, while Γ-Ctenitoxin-Pn1a and its derivatives as NMDA receptor modulators may be tested in in the direction of drugs improving cognitive functions and as such, supporting the treatment of schizophrenia. Argiotoxins, agatoxins and neurotoxins JSTX-3 as an NMDA-antagonist may be investigated in the context of a potential neuroprotective effect similar to that of the other NMDA-R blocker dizocilpine (MK-801) observed with long-term administration in the rat hippocampus (Cigel et al., 2021).

Interestingly, targeted injections of botulinum neurotoxin A (BoNT/A) into the human frontalis and procerus muscles results in a considerable reduction of depressive symptoms, 40–50% (Finzi & Rosenthal, 2014). The use of this toxin is considered safe and it can potentially be applied as a supporting method in the pharmacological treatment of depression. Noteworthy, antidepressive effect of a single dose of BoNT/A administered to a patient with major depressive disorder may persist for 4 weeks or more (Hexsel et al., 2013; Magid et al., 2014). The mechanism behind this effect is unknown, yet evidence obtained from several methodologically sound clinical trials encourage the use of this method of pharmacotherapy.

The neuroprotective effects of these neurotoxins or their derivatives could be exploited in the development of new drugs for the treatment of stroke, neurodegenerative diseases and the related cognitive deficits.

## Spider Neurotoxins and NMDAR-Related Glutamate Transmission

Up to 50 000 spider species has been discovered on Earth. Although, all of them possess functional venomous glands, only of 1400 venoms have been described so far. A wide spectrum of natural neurotoxic molecules isolated from spider venoms can target, often strongly and selectively, various types of neuronal receptors and ion channels. For instance hanatoxin (HaTx1) from *Grammostola spatulata* acts as selective blocker of voltage-gated Kv 2.1 potassium channel (Chen et al., 2012) but w-agatoxin 1A from *Agelenopsis aperta* inhibits P/Q calcium channels exclusively (Nakanishi, 2016). Several polypeptides, polyamines and acylpolyamines may act as more or less selective, noncompetitive antagonists of NMDAR-related glutamatergic signaling both in insects and vertebrates (Table 1). Intriguingly, number of spider neurotoxins affect insect cellular targets exclusively and their effect on vertebrate neurons is very subtle or none. For instance, a toxin peptide ω/κ-HXTV-Hv1a isolated from the venom of Australian Blue Mountains spider *Hadronyche versuta* blocks voltage gated calcium channels (Ca_v_) of the insect neurons with no effect on mammalian ion currents (King & Hardy, 2013).

**Table1:** An outline characteristics of the most important spider neurotoxins with a proven affinity to NMDA receptors

Neurotoxin name		Species	Molecular mass (Da)	Structure	Effects	Reference
Ctenitoxin-Pb48		*Phoneutria boliviensis* *Phoneutria nigriventer*	1341.5	Polypeptide	NMDAR antagonist	Estrada-Gomez et al. 2015
Ctenitoxin-Pb53		*Phoneutria boliviensis* *Phoneutria nigriventer*	1265.6	Polypeptide	NMDAR antagonist	Estrada-Gomez et al. 2015
Γ-Ctenitoxin-Pn1a	PnTx5(5–5)	*Phoneutria nigriventer*	5170	Polypeptide	NMDAR antagonist (100 nm)	Silva et al., 2016
δ-Ctenitoxin-Pn1a	PnTx4(6–1)	*Phoneutria nigriventer*	5838.8	Polypeptide	NMDAR antagonist (1 mM)	Lauria et al., 2020
Parawixin 10 (Pwx10)	PbTx1.2.3	*Parawixia bistriata*	587.5	Polyamine	EAAT2 blocker (10 ng/ml)	Fachim et al., 2015
Argiotoxin636 Argiotoxin659	ArgTX-636 ArgTX-659	*Argiope lobata* *Argiope aurantia* *Argiope lobata*	636.8 659.8	Polyamine Polyamine	NMDAR antagonist (3 mM) NMDAR antagonist (20 mM)	Albensi et al., 2000 Mueller et al., 1991
α-Agatoxin-489 Joro spider toxin	AG 489 JSTX-3	*Agelenopsis aperta* *Nephila clavata*	489.7 565.3	Polyamine Polyamine	NMDAR antagonist (20 mM) NMDAR antagonist (20 mM)	Monge-Fuentes et al., 2015 Mueller et al., 1991

## Γ-Ctenitoxin-Pn1a

The venom produced by South American *Phoneutria sp*. armed spiders is considered a source of toxins that can modulate NMDAR physiology. The frequency of serious bites caused by aggressive *Phoneutria nigriventer* species is relatively high therefore accumulating studies investigate the chemical composition of its venom. Over 40 neurotoxic molecules were identified and isolated from the crude secretion of spider venomous glands (Peigneur et al., 2018).

**Fig.1:**
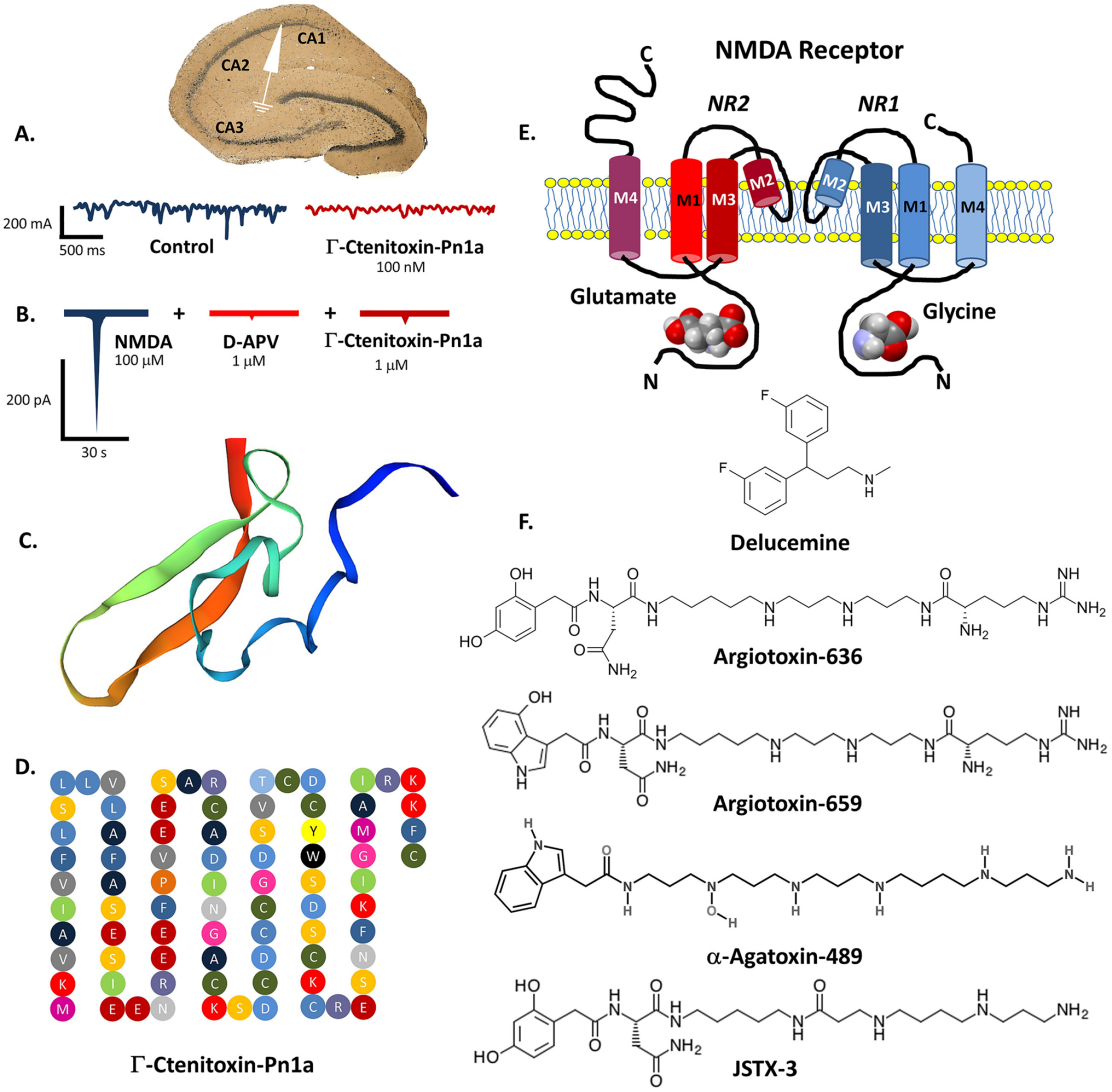
The whole-cell recording voltage-clamp of CA1 pyramidal neurons in hippocampal slices treated with Γ-Ctenitoxin-Pn1a (**A**). A decreased amplitude of NMDAR-mediated excitatory postsynaptic currents (EPSCs) is shown on the right (partly based on Silva et al. 2016, modified). Currents recorded from cultured rat hippocampal neurons (**B**). NMDA-evoked responses in the absence (control) and in the presence of Γ-Ctenitoxin-Pn1a or selective NMDA receptor antagonist—D-2-amino-5-phosphonovalerate; D-APV (data taken from Figueiredo et al., 2001, design modified). 3-D conformation of Γ-Ctenitoxin-Pn1a molecule (**C**) and its linear aminoacid sequence (**D**).The scheme of NMDA receptor molecule depicting all transmembrane domains of its two subunits GLUN1 and GLUN2 and ligand binding sites (**E**). Chemical structures of spider venom-derived polyamine NMDA receptor antagonists and antidepressant delucemine (**F**)

Γ-Ctenitoxin-Pn1a (Γ-CNTX-Pn1a) is a 81-aminoacid single-chain polypeptide with molecular mass of 5.17 kDa isolated from the PhTx4 fraction of the *P. nigriventer* venom (Fig. 1). Neurotoxin molecules are not able to cross blood–brain barrier (Oliveira et al., 2019). Intracerebroventicular injection of Γ-CNTX-Pn1a at dose 30 mg/individual did not cause behavioural impairment in mice. However, administration of 1 mM Γ-CNTX-Pn1a reversibly but selectively inhibits the NMDARs ion current in cultured rat hippocampal neurons and decreases by two-thirds the receptor response. Both AMPA and kainic glutamate receptors are not affected by the toxin (Figueiredo et al., 2001). Similarly, Γ-CNTX-Pn1a (100 nM) significantly reduces NMDARs activity in hippocampal slices stimulated via CA1 Schaffer collaterals (Silva et al., 2016). On the other hand, both peripheral (at dose 2.5–10 mg/ paw, itraplantar) and systemic (2.5 mg/kg) injection of Γ-CNTX-Pn1 decreases the L-glutamate and PGE2-induced hyperalgesia in rats (Oliveira et al., 2019, Lauria et al. 2020). The mechanism of neurotoxin action is not yet fully understood. Antinociceptive effects of Γ-CNTX-Pn1 may be caused by local Na_v_ 1.3 and Na_v_ 1.6 sodium channels blockade or/and inhibition of central NMDA-related signaling at the level of spinal dorsal horn neurons (Paiva et al., 2016). A distinct structural similarity (63% of sequence homology) with δ-Ctenitoxin-Pn1a may probably determine the neurophysiological properties of Γ-CNTX-Pn1. Interestingly, Γ-CNTX-Pn1 at picomolar concentrations exhibits neuroprotective effect on mouse corticostriatal neurons after glutamate-induced excitotoxicity and amyloid b-related cellular injury (Silva et al., 2016). Furthermore, in the mouse model of Huntington disease, Γ-CNTX-Pn1 (at dose 1 nM) promotes survival of BACHD neurons injured with toxic concentrations of glutamate (Silva et al., 2016). Novel neurotoxin ctenitoxin-Pb53, isolated recently from *Phoneutria boliviensis* venom is also considered as NMDARs modulator due to its high conformational homology with Γ-Ctenitoxin-Pn1a. Another factor identified in this venom, ctenitoxin-Pb48 structurally analogous with w-Ctenitoxin-Pn1a, may also potentially affect glutamate signaling (Estrada-Gomez 2015).

## Argiotoxins

Argiotoxin-636 (ArgTX-636, argiopine), C_29_H_52_N_10_O_6_ is an acypolyamine isolated from the venom of orb-weaver spiders *Argiope lobata* and *Argiope aurantia* (Fig. 1). Acting as very potent antagonist of NMDAR signalling, ArgTX-636 blocks receptor ion channel at low concentrations. ArgTX-636 at dose 3 mM acts as a NMDAR open channel blocker and it can halve the NMDARs activity of the rat cortical neurons in voltage-dependent manner. The similar effect was observed in cultured cerebellar granule cells and hippocampal neurons. Moreover, even lower concentrations of the neurotoxin are able to block tritium-labeled dizocilpine binding to the rat brain neuronal cell membranes. This effect is independent from alterations of glycine, glutamate and spermidine concentrations but a distinct affinity of ArgTX-636 to the Mg^2+^ binding site within NMDAR channel pore is suggested (Albensi et al., 2000). Mutations of a pore forming asparagine residue located in the NMDARs transmembrane M2 domain abolishes the ArgTX-636 action but sequence differences in the M2 region of both GluN2A and GluN2C subunits are not connected with the receptor sensitivity to ArgTX-636. Interestingly, ArgTX-636 has a very selective affinity for the GluN1/2A and GluN1/2B structural subtypes. This makes ArgTX-636 a valuable and precise molecular tool for the neuropharmacological study of NMDAR structure and function both in animal and in vitro models. Importantly, an inhibition of NMDAR transmission with ArgTX-636 as well as with other alkylamine toxins does not affect hippocampal LTP generation thus cognitive processes are not impaired (Albensi et al., 2000). Noteworthy, the structure of novel, preclinically studied selective serotonin reuptake inhibitor (SSRI) and NMDAR antagonist—delucemine (NPS-1506, Fig. 1.) is based on the ArgTX-636 molecule (Monge-Fuentes et al., 2015). Argiotoxin-659 (ArgTX-659, argiopinine), C_31_H_53_N_11_O_5_ is the next very potent NMDAR antagonist with analogous mode of action to ArgTX-636. Postsynaptic excitatory potentials of the rat hippocampal neurons are non-competitively silenced by both aforementioned neurotoxins at almost the same doses (20 and 24 mM respectively) (Mueller et al., 1991).

## α-Agatoxin-489 (AG 489)

Agatoxins are neuroactive polyamines isolated from the venom of desert grass spider *Agelenopsis aperta*. Three structural subclasses of agatoxins (a, m and w) grouping at least 16 isoforms are currently known. Majority of them such as aforementioned w-agatoxin 1A exhibit potent and very selective affinity to neuronal ion channels. α-Agatoxin-489, (*N*-(20-Amino-4-hydroxy-4,8,12,17-tetraazaicosan-1-yl)-2-(9*H*-purin-3-yl)acetamide, C_26_H_47_N_7_O_2_, Fig. 1) is the most potent non-competitive blocker of NMDAR-dependent calcium current in rat cerebellar granule cells and hippocampal neurons (Kiskin et al., 1992), however other a-agatoxins also exhibit analogous inhibitory activity. Noteworthy, AG489 unlike ArgTX-636 enhances [^3^H]-dizocilpine binding to rat brain neuronal membranes at low concentrations only via stimulation of NMDARs polyamine site (Monge-Fuentes et al., 2015).

## Joro Spider Toxin (JSTX-3)

Studies on neurotoxin JSTX-3, C_27_H_47_N_7_O_6_ (Fig. 1) isolated from the venom of Joro spider *Nephila clavata* have provided unconclusive results. On the one hand, it is considered an inhibitor of EPSPs in rat hippocampal CA1 neurons at dose 20 mM (Mueller et al., 1991) but other reports suggest that only minor part of ion current blockade by JSTX-3 is an effect of NMDARs inhibition (Sahara et al., 1991) or neurotoxin activity is rather weak, as in case of rat spinal neurons (Jones & Lodge, 1991).

## Parawixin 10 (Pwx10)

Parawixin 10 (Pwx10, PbTx1.2.3) a polyamine isolated from the venom of South American species *Parawixia bistriata* modulates glutamatergic signaling indirectly via stimulation of glutamate transporter 2 (EAAT2) activity. Studies on rat cortical synaptosomes in vitro revealed an increase of neurotransmitter reuptake after treatment with Pwx10 at dose 10 ng/ml without disturbing of EAAT2 affinity to sodium ions. The precise mechanism of Pwx10 action is so far unknown, however it should be suggested that it may potentially reduce excitotoxic injuries without blocking NMDARs action (Fachim et al., 2015).

## Concluding Remarks

The NMDA receptor modulators are currently an important subject of widespread neuropharmacological research. Spider polypeptide and arylkylamine neurotoxins act as potent and selective NMDAR antagonists that do not disturb central cognitive mechanisms related to hippocampal glutamate transmission, LTP generation and synaptic plasticity. It is possible that such promising neuroactive peptides can be clinically applicable in a wide spectrum of neuropsychiatric disorders, including epilepsy, neurotrauma and ischemic injuries. Several contemporary research suggest an intriguing possibility of spider-venom derived drug designing. These novel medications can potentially be helpful in the treatment of stroke and neurodenerative diseases. For instance, neurotoxin Hi1a isolated from the Australian species *Hadronyche infensa* exposes distinct neuroprotective properties and significantly reduces post ischemic brain dysfunctions in animal model (Chassagnon et al., 2017). Unfortunately, number of these macromolecular compounds particularly *Phoneutria sp*. neurotoxins do not cross blood–brain barrier and their efficient delivery to the brain is therefore extremely difficult. Nevertheless, many of them may be treated as structural scaffolds or model molecules in the development of novel, potentially effective and more safe pharmacological strategies.
